# Textural and physicochemical predictors of sensory texture and sweetness of boiled plantain

**DOI:** 10.1111/ijfs.14765

**Published:** 2020-08-30

**Authors:** Hermann Antonin Kouassi, Emma Fernande Assemand, Olivier Gibert, Isabelle Maraval, Julien Ricci, Deless Edmond Fulgence Thiemele, Christophe Bugaud

**Affiliations:** ^1^ Department of Food Sciences and Technologies Laboratory of Food Biochemistry and Technology of Tropical Products University Nangui Abrogoua 02 BP 801 Abidjan 02 Ivory Coast; ^2^ CIRAD UMR QUALISUD F‐34398 Montpellier France; ^3^ QUALISUD Univ Montpellier CIRAD Montpellier Institut Agro Univ d’Avignon Univ de la Réunion Montpellier France; ^4^ Department of Biochemistry ‐ Genetics University PELEFORO GON COULIBALY BP 1328 Korhogo Ivory Coast

**Keywords:** Musa, cultivar, sensory profile analysis, penetrometry, texture profile analysis, multilinear regression

## Abstract

Boiled pulp is a major form of consumption for plantain. We assessed instrumental (puncture test and texture profile analysis) and sensory texture attributes of 13 plantain cultivars, two cooking hybrids and one dessert banana at different stages of ripeness after cooking in boiling water. Firmness, chewiness, stickiness, mealiness, sweetness and moistness described sensory variability, which was greater between stages of ripeness than between types of cultivars. Firmness and chewiness were well‐predicted by instrumental force and hardness (*r* ^2^ > 0.72), and by soluble solid and dry matter content (*r* ^2^ > 0.85). Complementary sensitivity analysis revealed that a pulp puncture force or a hardness of at least 2.1 N or of 0.3 N/mm^2^ was needed before a difference in firmness or chewiness could be perceived; a Brix of 3.7 was required to ensure a detectable difference in sweetness. Rheological and biochemical predictors can be useful for breeders for high‐throughput phenotyping.

## Graphical abstract

Prediction of firmness by puncture force for boiled plantain

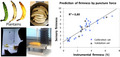

## Introduction

Plantain is a staple food in many tropical areas, including in West and Central Africa, with between 120 and 150 plantain varieties reported (Lassoudière, 2010; Tomekpe *et al*., 2011). More than 20 recipes are mentioned using plantains and other cooking bananas (Coulibaly, 2008). Irrespective of the continent, boiling in water is the most widely used method of preparing plantain for consumption (Dufour *et al*., 2009; Kouassi *et al*., 2020). Plantain can be consumed at different stages of ripeness: boiled at a green stage like other starchy food (e.g. potato, yam and cassava) or eaten at a ripe/overripe stage like a fruit (Tshiunza *et al*., 2001).

Household studies based on surveys and consumer tests have highlighted some preferences or the non‐acceptance of some plantain varieties at a given stage of ripeness, and for a given use (Ekesa *et al*., 2012; Kouamé *et al*., 2015). In eastern Ivory Coast, some local varieties including Olaya (French), Banadiè and Ameletia (False Horn) are very popular when ripe and boiled to make *foutou* (Kouassi *et al*., 2020). Agronomic yield and technological performance (easy‐to‐peel, pulp‐to‐peel ratio and cooking ability) are considered as important criteria by producers and processors, but the main criterion for consumers is sensory quality. Although texture and flavour are claimed to be the main criteria for plantain acceptability (Dzomeku *et al*., 2006; Assemand *et al*., 2012), almost no data are available that objectively describe the sensory quality of boiled plantain assessed by a trained panel and measured by instrumental analyses. As reported in the literature, great care has to be paid to the temperature at which the boiled pulp is evaluated by panellists, since cooling after cooking greatly affects textural properties, especially firmness (Voisey *et al*., 1978; Leighton *et al*., 2010). It is thus worth investigating the use of instrumental measurements to identify predictive sensory traits expected by consumers of plantain. Rapid implementation of instrumental tests could enable earlier selection of new hybrids in breeding programmes.

Data on the sensory diversity of boiled plantain are seldom available, and those that are, only concern a limited number of cultivars using hedonic characterisation (Assemand *et al*., 2012; Belayneh *et al*., 2014). The lack of sensory characterisation may be explained by the difficulty involved in accessing a wide range of cultivars with contrasting sensory traits, controlling plantain harvest and ripening conditions, and the conditions of tasting after cooking.

However, a number of uniaxial puncture, compression and double‐compression tests have been developed to assess plantain pulp texture (Peleg & Gómez Brito, 1977; Kajuna *et al*., 1997; Qi *et al*., 2000; Gibert *et al*., 2010). With the exception of texture profile analyses, most protocols focused on the firmness component, often at only one ripening stage, and have not been correlated with objective sensory attributes.

The aims of the present study were thus to (i) describe the sensory diversity of boiled plantain at three apparent contrasting stages of ripeness (green, half‐ripe and ripe) and (ii) predict sensory attributes of texture and taste using textural and physicochemical characterisation.

## Materials and methods

### Plant material, sampling and controlled ripening

Bunches were sourced from sixteen genotypes of banana (*Musa spp*.). Bunches from twelve plantain and two hybrid (AAAB) varieties were collected at the mature green stage at Krindjabo in south‐eastern Ivory Coast (5°24N; 3°13W) and Azaguié (5°38N; 4°05W) (Table 1). After packing, fruit samples were dispatched by air to CIRAD Montpellier at the earliest possible opportunity and stored at 14 °C until use. Bunches from one plantain cultivar (AAB) Dominico Harton from Honduras and from one Cavendish dessert type cultivar (AAA) from Ecuador were purchased from a local fruit and vegetable store in Montpellier (France) at the mature green stage and also stored at 14 °C until use.

**Table 1:** List of cultivars for the study

Vernacular name	Scientific name	Type*	Genotype	Collection area/Country	Abbreviation	Grade (mm)	Ripening stage (quantity of products)		
							MG (11)	HR (16)	FR (8)
Cultivars used for calibration									
Afoto	‐	FH	AAB	Kri/CI	af	39	‐	1	1
Agnrin	Light French	F	AAB	Kri/CI	ag	29	‐	1	1
Alosso	Horn 1	FH	AAB	Kri/CI	al	40	‐	1	‐
Big ebanga	Big ebanga	FH	AAB	Aza/CI	be	32	1	‐	‐
Corne 1	Horn 1	FH	AAB	Aza/CI	co	39	1	1	1
Dechair	‐	FH	AAB	Kri/CI	de	29	1	1	1
Kaki	Horn 5	FH	AAB	Kri/CI	ka	29	‐	2	‐
Kpatregnon	‐	RH	AAB	Kri/CI	kp	31	3	‐	‐
Molegna	‐	F	AAB	Kri/CI	mo	24	‐	1	1
N’gretia	French round tip	F	AAB	Kri/CI	ng	24	‐	1	1
Orishele	Orishele	FH	AAB	Aza/CI	or	34	1	1	1
Saci	Saci	F/FH	AAB	Aza/CI	sa	32	1	1	1
Cultivars used for validation									
FHIA 21	FHIA 21	Hyb	AAAB	Aza/CI	fh	33	‐	1	‐
PITA 3	PITA 3	Hyb	AAAB	Aza/CI	pi	34	‐	1	‐
Dominico Harton	Dominico harton	FH	AAB	Mpl/Fr	dh	38	2	3	‐
Cavendish	Cavendish	D	AAA	Mpl/Fr	ca	30	1	‐	‐

* type: FH: False horn; F: French; RH: real Horn; F/FH: intermediate between French and False horn; Hyb: Hybrid; D: Dessert; Kri/CI: Krindjabo/Côte d’Ivoire; Aza/CI: Azaguié/Côte d’Ivoire; Mpl/Fr: Montpelier/France; MG: mature green; HR: half ripe; FR: fully ripe

Fruits were judged to be at the mature green stage (MG), if there was no visible discoloration of the peel. Prior to storage at 20 °C with 80% humidity until used for cooking and sensory tasting, two‐thirds of the mature green fruits of each cultivar underwent ethylene treatment (1 mL L^−1^) for 24 h to trigger the ripening process. One‐third of the mature green fruits ripened during for 4 days. Fruits presented a peel colour more yellow than green, which correspond to a half‐ripe (HR) stage of ripeness, according to Giami & Alu (1994). One‐third of the mature green fruits were stored for 8 days. Fruits exhibited an apparent yellow peel colour, which correspond to a fully ripe (FR) stage of ripeness. Most varieties could not be assessed at all stages of ripeness, due to early ripening or inadequate availability of fruits. Eleven mature green, sixteen half‐ripe and eight fully ripe samples were evaluated by panel (Table 1).

### Cooking

After washing and hand‐peeling, banana pulps were immediately cooked in a large volume of boiling water (100 °C at 50 m above sea level in Montpellier, France) in individual stainless‐steel pans (6:1 tap water‐to‐banana ratio), in a dedicated room. The cooking time was adapted to the stage of ripeness of the fruits, known traditional culinary habits (Gibert *et al*., 2010; Kouassi *et al*., 2020), and some preliminary cooking trials. Cooking time was set to 10 min for fully ripe, 20 min for half‐ripe and 30 min for mature green plantains.

### Sensory analysis

#### Preparation of samples and tasting service

At each cooking time, whole boiled plantains were removed from the water and dried superficially for less than 30 s using a soft absorbent tissue. After the apical and peduncle ends were removed, the pulp was cut into 3‐cm sections. Each section was placed in a porcelain dish prior to being randomly served to the panellists at the desired time. Given the above‐mentioned influence of temperature on the sensory perception of firmness, we set up a tasting protocol to ensure that all the panellists assessed the products at exactly the same target temperature. A K‐type thermocouple was immediately inserted to the geometrical core of a reference sample of each boiled product. The temperature was monitored throughout the cooling process using an Almemo 2690‐8A data logger (Ahlborn GmbH, Germany), placed in an individual box in the sensory laboratory which was in the immediate proximity of the cooking room. When the temperature of the reference sample dropped to 60 °C, a signal was given to the trained panellists to start tasting the samples, using an evaluation protocol and a scaling rate for each attribute (Table 2). An average reference temperature of 50 °C was recorded when the panellists finished evaluating a product. Before tasting a new product, panellists were required to rinse their mouth with mineral water. The samples were coded with an anonymous random 3‐digit number and were served monadically. Thirty‐five products were assessed in six sessions, six products being judged in each session. The relative humidity and temperature of the sensory laboratory were also kept constant at 21+/‐ 1°C and 24+/‐ 5% RH (AFNOR, 2011).

**Table 2:** Sensory attributes of banana boiled

Attribute Family	Attribute	Definition	Evaluation protocol	Rating scale
Sensory Texture	Firmness	Force required to obtain deformation, penetration or rupture of the banana	Put in the mouth a piece of banana and Evaluate the force necessary to obtain the deformation of the product between the teeth during the first compression	0: Soft
				10: Firm
	Chewiness	Energy or number of chews necessary to chew the banana to make it ready to be swallowed	Place the sample in the mouth, chew it at the rate of one chewing per second and assess the number of chews before swallowing (NB: chew the same amount of banana)	number of chews
	Mealiness	Mechanical property linked to cohesion and the presence of fine particles in the product during chewing	Put a piece of banana in your mouth and assess the presence of mealiness particles during chewing	0: Low
				10: Strong
	Stickiness	Force required to peel off the fraction of product adhering to the interior of the oral cavity	Press a piece of banana between the molars and appreciate the adhesion of the product	0: Low
				10: Strong
Taste	Sweetness	Elemental flavour caused by dilute aqueous solutions of various substances such as sucrose or aspartame	Put a piece of banana in your mouth, chew it and swirl it around your tongue to detect the sweet flavour	0: Low
				10: Strong
Mouth impression	Moist	Perception of the amount of water absorbed or released by the product	Once the flavours are detected, moisture is felt by turning the product in the mouth	0: Dry
				10: Moist

#### Production of the assessment vocabulary and panel training

Quantitative descriptive analysis was used for sensory evaluation by 13 trained panellists (five women and eight men, aged between 21 and 60 years old) on the CIRAD Montpellier panel. Subjects were screened and selected for their sensory ability, their willingness to eat boiled bananas and their availability for the investigation. First, the panellists defined a descriptive vocabulary for boiled banana, which previously did not exist in the literature. Five different samples (different varieties and different stages of ripeness) were provided. Panellists were asked to list the sensory characteristics that they considered important to describe the samples. An additional session was needed to choose the pertinent attributes, agree on their definition and on the evaluation protocol (Table 2). Finally, after the training sessions, six descriptors were selected: four for texture in the mouth (chewiness, firmness, stickiness and mealiness), one for taste (sweetness) and one for impression in the mouth (moistness). Given the difference in the perception of chewiness among the members of the panel, this attribute was the subject of a specific evaluation, in which the panellists were asked to chew a sample, equivalent to a slice approximately 1 cm thick and 3 cm in diameter. The panellists recorded the number of times they chewed before swallowing. As the number of chews varied greatly from one panellist to another, this number was computed in a non‐dimensionalised form for each panellist, then converted to a value between 0 and 10 (0 being the lowest dimensionless value of all products and all panellists, and 10 the highest). Other textural attributes were described by firmness, mealiness and stickiness, taste by sweetness, and sensation in the mouth by moistness. These attributes were sorted on a discrete scale ranging from 0 (very weak) to 10 (very strong).

Four sessions were needed to train panellists and to assess their performance based on three criteria: repeatability, agreement within the panel and discrimination. Repeatability was considered effective for an attribute if the deviation between two identical products was equal to or less than 2. Agreement was considered to be reached if 70% of the deviation (absolute value) between the panel average and the average of each panellist (for an identical product assessed twice) was lower than the standard deviation of the panel. The ability to discriminate is generally assessed by means of the product effect of an analysis of variance model. The product F values associated with the product effect reflect the ability to discriminate. Finally, a panellist performs well in terms of repeatability and agreement with panel, if he or she is repeatable and in agreement on at least 70% of the attributes. No rules were established for the discrimination criterion but the panel facilitator talked to panellists who had difficulty using the rating scale to distinguish the products, especially the attributes the panel succeeded in distinguishing.

### Instrumental measurement of texture

Instrumental parameters of the texture of boiled pulp were measured by puncture and by double‐compression tests using two identical TAX‐T2 texture analysers (Stable Micro Systems, Ltd., Surrey, UK). Measurements were made on samples taken from the same boiled banana pulp. Immediately after cooking, and removing superficial water with a soft tissue, the apical and peduncle ends of the samples were removed, and the remaining pulp of each sample was divided into two equivalent portions to conduct the two tests. All textural measurements were made at temperatures set at 50 and 60 °C. The temperature during pulp cooling was monitored by the Almemo 26‐908A data logger equipped with a K‐type thermocouple (diameter 1 mm) inserted into the geometrical core of each sample.

For the puncture test, a 5‐mm‐diameter cylindrical metal borer (surface area ~ 20 mm^2^) penetrated the banana pulp at a constant speed (1 mm s^−1^) to a depth of 15 mm. The maximum force applied during the measurement was recorded.

Two cylindrical samples (30 mm in length) were removed from the remaining half‐portion of fruit pulp along the longitudinal axis for the double‐compression test performed using Texture Profile Analysis (TPA). Two compression cycles, each corresponding to 20% of sample strain, were performed at a constant crosshead speed of 1 mm s^−1^, using a probe 60 mm in diameter. Force–time curves were recorded by the software of the instrument. The TPA parameters were computed as per Szczesniak (1963). Since the instrumental textural parameters took the real surface of the sample into account, the surface area of the banana section was then estimated from a slice made in the median of banana that was photographed and processed with ImageJ software version 1.52*k* (NIH, USA). Hardness (N mm^−2^) refers to the peak force during the first bite divided by the surface area of the banana section. Cohesiveness (with no unit) is represented by the ratio of the area under the second bite to the area under the first bite. Adhesiveness (mN.s mm^−2^) is defined as the negative force area (divided by the surface area of the banana section), and springiness (with no unit) is represented by the ratio of the distance travelled during the first descent to the distance travelled during the second descent of the probe.

### Chemical parameters

Dry matter content was determined by thermogravimetry, using 2 g (fw) of samples at 70 °C until constant weight. Soluble solid contents were measured by refractometry after dilution of banana puree in an equal volume of Milli‐Q water and filtration. Titratable acidity was determined by diluting 3 g (fw) of banana puree in 30 mL of Milli‐Q water, and titrating with 0.1 N NaOH to endpoint at pH 8.1 using an automatic Titroline 96 titrator (Schott‐Geräte GmbH, Germany) as per AOAC (1995).

### Statistical analysis

XLSTAT software, version 2020.1.1.64570, was used for statistical analysis. ANOVA was performed (only on four cultivars at all stages of ripeness) to determine significant differences between stages of ripeness. A calibration set was made using 12 varieties (27 samples) at different stages of ripeness to build a linear discriminant analysis (LDA) prediction model for the classification of the sensory attributes; four cultivars (eight samples) were later used as the validation set. Principal component analysis (PCA) was performed to describe the sensory characteristics of the boiled plantain. As the sensory scales were the same for all attributes, the matrix covariance was used for sensory data (Borgognone, Bussi & Hough, 2001). Simple and multiple linear regressions (stepwise type) were performed to predict sensory attributes with the instrumental parameters (puncture and TPA) recorded at 50 and 60 °C and with biochemical parameters after cooking. The probability of entering a predictor in the model was 0.05 and of removing it, 0.1.

Regression models were calibrated with 27 samples of plantains from Ivory Coast and validated with eight samples of other varieties (FHIA 21, PITA 3, Cavendish, Dominico Harton). Calibration performances were evaluated with coefficients of determination (*R* ^2^) between predicted and observed variables, and the root mean square error of calibration (RMSEC):

$$\text{RMSEC}=\sqrt{\frac{\sum_{i=1}^{n}{\left(Yi_{p}‐Yi_{o}\right)}^{2}}{n}}$$

where *Yip* and *Yio* are, respectively, the predicted and observed values for sample *i*, *n* is the number of samples (n = 27). The robustness of validation was evaluated by the root mean square error of validation (RMSEV) that was calculated with a similar expression to that of RMSEC with *n *= 8. According to Hellerstein *et al*. (2004), 95% of new observations are between ± 2xRMSEC.

A sensitivity analysis was performed according to Harker *et al*. (2002a) to assess the performance of the panel represented by a minimum perceptible difference, to a given variation of the instrumental parameter (*P* < 0.1). The minimum difference (∆j) was calculated for each prediction parameter in the regression obtained from the calibration data:

$$\Delta j=\frac{t_{0,9}s\sqrt{2}}{a_{j}}$$

where *t_0,9_* is the 90th percentile of t‐distribution with 2 degrees of freedom (*n *= 27 in this study), *s* is the root mean square errors in the regression, *aj* is the coefficient of the predictive parameter *xj* in regression $y=b+\sum_{j=1}^{p}a_{j}x_{j}$; *p* is number of predictors in regression (*P* ≤ 2) and b is the constant. This sensitivity analysis was performed if *R* ^2^ > 0.5.

## Results and discussion

### Physicochemical and sensory characteristics of boiled plantains

#### Chemical characteristics of boiled plantains

Significant differences in dry matter content, soluble solid contents and titratable acidity of boiled plantain (Table 3) were observed between ripeness stages. The 13% decrease in dry matter content during ripening was certainly more linked to the decrease in this parameter in raw fruits than to an increase in water absorption during cooking (Ahenkora *et al*., 1997). The increase in soluble solids and titratable acidity in boiled products during ripening is also hypothesised to reflect those of raw fruits (Ahenkora *et al*., 1997). Some soluble solids could also result from starch hydrolysis. Further studies are needed to evaluate the real contribution of the chemical composition of raw fruits and of the changes that occur during cooking to the composition of the boiled product.

**Table 3:** Physicochemical parameters of boiled plantain according to ripening stage

Chemical parameters	Mature green stage	Half‐ripe	Full‐ripe
Dry matter content (g/100g FW)	35.39 ^a^ ± 1.95	32.22 ^b^ ± 0.26	30.65 ^b^ ± 0.14
Soluble solid content (°Brix)	2.30 ^c^ ± 0.38	8.07 ^b^ ± 0.81	12.03 ^a^ ± 0.66
Titratable acidity (meq/100g FW)	2.06 ^b^ ± 0.82	7.02 ^a^ ± 0.96	7.23 ^a^ ± 1.02

For each parameter, means with different letters along the same row are significantly different at *P* < 0.05 (Tukey’s test).

Chemical variability was observed between banana genotypes at a given stage of ripeness. The lower dry matter content in dessert bananas and cooking hybrids is in line with the results of previous studies (Gibert et al., 2009). Saci had the highest dry matter content at the mature green stage (38.6 g/100g FW), Alosso at the half‐ripe stage (36.3 g/100 g FW) and Afoto at the ripe stage (32.6 g/100g FW). It is interesting to note that some cultivars had about 30 g/100g FW of dry matter content at the full‐ripe stage, which could be a valuable asset for some uses (Dadzie & Orchard, 1994). At the full‐ripe stage, Saci was the plantain with the highest soluble solid contents (13.1° Brix) and the lowest titratable acidity (6.3 meq/100g FW), as well as being one of the sweetest cultivars evaluated.

#### Instrumental texture of boiled plantain

Except for hardness and puncture force (*R* ^2^ = 0.84, 0.86, respectively), less significant correlations were obtained when other textural parameters were compared at 50 and 60 °C, including springiness, cohesiveness and adhesiveness (*R* ^2^ = 0.60, 0.38 and 0.23, respectively). These results suggest that adhesiveness and cohesiveness traits are sensitive to slight structural variations in the fruits. Higher hardness and puncture forces at 50 °C than at 60 °C (3% and 7%, respectively) appeared to confirm the influence of cooling on the sensory perception of cooked products (Voisey *et al*., 1978; Leighton *et al*., 2010).

Except for adhesiveness, the other instrumental parameters decreased significantly during ripening (Table 4). Cooking time decreased with increasing ripeness, and hardness and puncture force were 40% lower at the half‐ripe stage and 80% lower at the full‐ripe stage than at the green stage. This implies that the stage of ripeness strongly contributes to changes in texture. As reported earlier, the softening of banana pulp during ripening is mainly linked to granular deterioration of the starch (Shiga *et al*., 2011). Thus, the increase in firmness on cooling may partially offset the marked textural loss that occurs during starch hydrolysis, when optimal cooking conditions were assessed at different ripening stages.

**Table 4:** Instrumental parameters of texture of boiled plantain according to ripening measured at 50 and 60 °C

Instrumental texture parameters	Measured at 60°C			Measured at 50°C		
	Mature green	Half ripe	Full ripe	Mature green	Half ripe	Full ripe
Hardness (N/mm^2^)	0.79 ^a^ ± 0.23	0.36 ^b^ ± 0.08	0.13 ^b^ ± 0.03	0.91 ^a^ ± 0.14	0.50 ^b^ ± 0.09	0.13 ^c^ ± 0.05
Adhesiveness (mN.s/mm^2^)	7.25 ^a^ ± 2.98	15.73 ^a^ ± 7.07	8.51 ^a^ ± 2.01	15.60 ^a^ ± 1.98	16.99 ^a^ ± 5.07	9.88 ^a^ ± 4.91
Cohesiveness	0.66 ^a^ ± 0.05	0.53 ^a^ ± 0.08	0.62 ^a^ ± 0.01	0.68 ^a^ ± 0.05	0.56 ^b^ ± 0.02	0.62 ^ab^ ± 0.04
Springiness	0.76 ^a^ ± 0.03	0.64 ^b^ ± 0.02	0.66 ^b^ ± 0.05	0.77 ^a^ ± 0.01	0.64 ^b^ ± 0.02	0.68 ^b^ ± 0.02
Puncture force (N)	4.62 ^a^ ± 1.06	2.15 ^b^ ± 0.27	0.77 ^b^ ± 0.16	5.11 ^a^ ± 1.19	3.02 ^ab^ ± 0.47	0.89 ^b^ ± 0.41

For each parameter, means with different letters along the same row are significantly different at *P* < 0.05 (Tukey’s test).

Varietal differences in instrumental texture were less marked than differences between the different stages of ripeness. As expected, the dessert banana cultivar (Cavendish) and the hybrid FHIA 21 displayed lower hardness and puncture force after cooking than plantains, confirming their greater sensitivity to heat (Gibert *et al*., 2010). French plantains (Agnrin, N’gretia and Molegna) showed the greatest firmness at the mature stage. Indeed, it is known that French plantains ripen more slowly than other plantains (Loa *et al*., 2017), and the fact that the cultivars Agnrin and Molegna give a tender and firm foutou at the ripe stage could reflect their high post‐cooking firmness, as reported by Assemand *et al*. (2012) and Kouassi *et al*. (2020).

#### Sensory traits of boiled plantain

**Figure 1:**
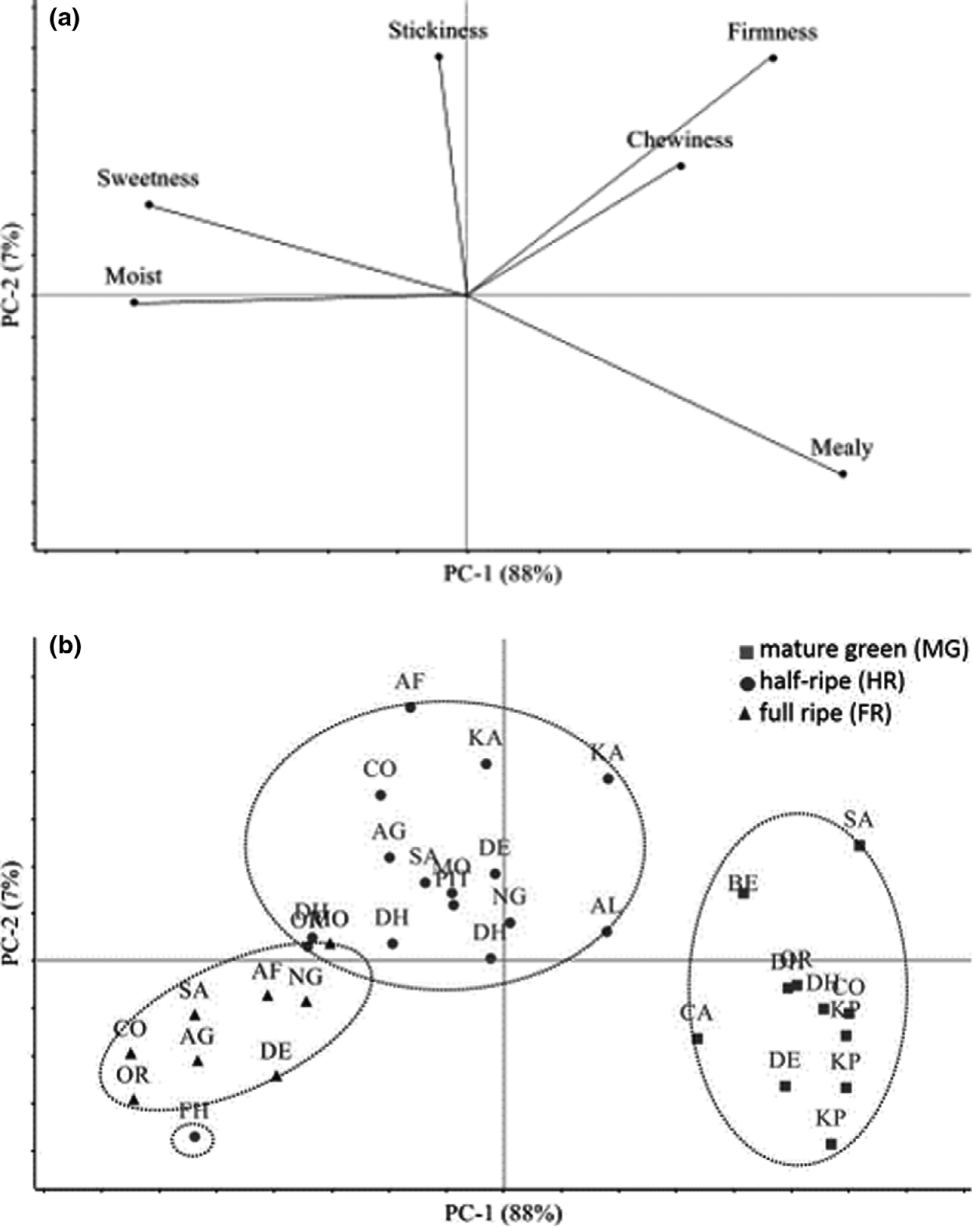
(a) Principal Components plot of the first two PC loading vectors. Sensory textural attributes: moist, sweetness, stickiness, firmness, chewiness and mealiness. (b) Principal Components plot of the first two PC score vectors. Varieties: AF, afoto; AG, agnrin; AL, alosso; BE, big ebanga; CA, cavendish; CO, corne 1; DE, dechair; DH, dominico harton; FH, fhia 21; KA, kaki; KP, kpatregon; MO, molegna; NG, n’gretia; OR, orishele, PI, pita 3; SA, saci, at mature green stage (MG), half‐ripe stage (HR), and full ripe stage (FR).

With the linear classification, the LDA calibration model correctly classified 100% of the varieties according to their stage of ripeness (with eight green, twelve half‐ripe and six ripe samples) using the sensory attributes (data not shown). Except the half‐ripe hybrid cooking sample FHIA that was classified as ripe, the complementary validation set correctly classified the three plantains Corne 1, Dechair and Orishele at all stages of ripeness based on some distinguishable sensory attributes. Unexpectedly, such supervised classification at different stages of ripeness also suggested some differences among varieties. Complementary PCA using the covariance as the index of similarity reflected the different sensory traits of boiled plantain. The first principal component (PC1), which represented 88% of total variance, opposed firmness, mealiness and chewiness to sweetness and moistness (Fig. 1a). Such variation (*R* ^2^> 0.80) in sensory attributes (hardness, firmness, springiness and mealiness) was already observed by Thybo & Martens (1999) in boiled potato. In peach, fruits with high mealiness are associated with poor juiciness (Infante *et al*., 2009), which can be considered as the equivalent of an impression of moistness in the mouth. The distribution of the samples on the PC1 axis clearly separated varieties according to their stage of ripeness (Fig. 1b). The mechanical properties, that is texture (firmness, chewiness) and the geometrical properties (mealiness) of boiled plantains decreased with increasing ripeness, whereas moistness and sweetness increased with ripeness. The second principal component accounted for 7% of PCA variance and designated stickiness. Boiled plantains were perceived as stickiest at the intermediate stage of ripeness. The three stages of ripeness were clearly differentiated, suggesting an inter‐cultivar sensory variability closely correlated with ripening.

At an equivalent stage of ripeness, sensory differences were observed between banana types and cultivars. As expected, the dessert type banana (Cavendish) was found to be less firm and moister than plantains at the mature green stage. Due to lower mechanical strength, at the half‐ripe stage, the hybrid FHIA 21 actually behaved like full‐ripe plantains. Despite its interesting agronomic characteristics and attractive yield (Vawa *et al*., 2016), in the present study, this hybrid displayed poorer cooking ability than plantains at the green stage (Gibert *et al*., 2010) especially when ripe. The other hybrid PITA 3, which is disease resistant and has good agronomic capacity (Vawa *et al*., 2016), had similar sensory characteristics to plantains at the half‐ripe stage, confirming the results of previous studies (N’da, 2016). Some of the plantains were distinguishable based on their textural traits at given stages of ripeness, such as Saci (intermediate cultivar between French and False horn) and Kpatregnon (real horn) at the mature green stage, Afoto and Corne 1 (both False horn) at the half‐ripe stage and finally, Corne 1, Orishele (False horn), Moligna (French plantain) and Saci at the full‐ripe stage.

### Predicting sensory traits using physical–chemical parameters

#### Predicting texture attributes with instrumental texture parameters

The sensory texture of boiled plantains regressed against instrumental texture parameters led to a better prediction model at 50 °C than at 60 °C (Table 5). Firmness, chewiness, mealiness and moistness were well predicted by instrumental texture parameters (*R* ^2^> 0.76, RMSEC < 1.0, and RMSEV < 1.2). The best prediction of chewiness (*R* ^2^ = 0.84, RMSEC = 0.5 and RMSEV = 0.6) was most probably due to the way this attribute was evaluated. Indeed, unlike the other attributes, it was assessed by recording the number of chews, which the panellists found easier than scoring on a 0–10 scale. This objective method is easily reproducible and applicable to other food products. To our knowledge, this is the first time that texture attributes, especially firmness, have been predicted by instrumental texture parameters in boiled plantain. These results are in agreement with those of Thybo *et al*. (2000), who concluded that uniaxial compression parameters are good predictors of the mechanical sensory properties of hardness, firmness and springiness in boiled potato. Overall, the good predictions reflect the good performance of the panel and good mastery of the methodological strategy. Indeed, the temperature of the samples was well‐controlled in both the sensory and instrumental measurements, which has seldom been applied to cooked foods. The use of contrasting varieties cooked at three different ripening stages made it possible to fully use the sensory scale, and thus promoted high correlations.

**Table 5:** Prediction of texture sensory texture with instrumental parameters measured at 50 °C

Attributes	Regression equation	Performance of calibration		Validation robustness	Sensitivity analysis
		*R* ^2^	RMSEC	RMSEV	
Firmness	Y = 1.48 + 1.10*Fp Y = 1.85 + 6.06*Hardness	0.80 0.72	0.9 1.0	0.8 0.9	2.05 N 0.41 N mm^−2^
Chewiness	Y = 1.36 + 0.74*Fp Y = 1.39 + 4.52*Hardness	0.77 0.84	0.6 0.5	0.6 0.6	2.08 N 0.30 N mm^−2^
Stickiness	Y = 6.87 + 59.01*Adhesiveness – 4.92*Springiness	0.32	0.7	1.0	‐
Mealiness	Y = −11.28 + 4.46*Hardness + 17.64*Springiness	0.82	1.0	1.2	0.56 N mm^−2^
Moist	Y = 7.53–6.48*Hardness	0.76	1.0	0.6	0.39 N mm^−2^

Calibration samples = 27; Validation samples = 8; Fp: puncture force. The sensitivity analysis provided the minimum instrumental difference required to ensure a perceptible sensory difference (*P* < 0.1). The table lists the difference only for the first prediction parameter and only for the best predictions.

As expected, hardness measured by compression or puncture satisfactorily predicted firmness, defined as the force required to obtain deformation, penetration or rupture of the pulp (Table 2). Both instrumental parameters expressed the strain during the first chew. Their relationship with chewiness was unexpected, since it rather designates a number of chews, or the energy required to masticate the product until it reaches a suitable state for swallowing. The predictions of mealiness and moistness by hardness, and to a lesser extent of springiness, were even more unexpected. As the firmest fruits were also the mealiest and the least moist, these predictions could be due to collinearity between these sensory traits. Stickiness was expected to be predicted by adhesiveness, but once again, this attribute was badly predicted (*R* ^2^ = 0.32) as previously observed in dessert banana (Bugaud *et al*., 2013) and boiled potatoes (Thybo & Martens, 1999). On the one hand, this could be explained by the difficulty for panellists to score the entire scale of stickiness. On the other hand, the instrumental method is probably not the best way to assess this criterion. As proposed by Kojima *et al*. (1994), a relaxation test may be more suitable than puncture or compression tests.

Sensitivity analysis revealed how big a difference in the instrumental parameter was needed to cause a detectable difference (*P* < 0.1) in the sensory attributes of boiled plantain (Harker *et al*., 2002b). Sensitivity analysis showed that a difference of 2.0 N in the puncture force is required for panellists to perceive a difference in firmness between two products (Table 5). This value was twice that found by Bugaud *et al*. (2013) in dessert bananas using the same probe. The higher perception threshold in our study is mathematically explained by the coefficient of predictive parameter (1.10) which was twice lower than in the above‐mentioned study on dessert bananas (2.23). We hypothesised this might be due to some differences in the responses of different trained panels, or in the variability of textural attributes among dessert bananas (as per Bugaud *et al*., 2013) and current plantains from Ivory Coast. Differences of 0.41 or 0.30 N mm^−2^ in hardness led to a noticeable difference in firmness and chewiness, respectively. Based on the results of the sensitivity analysis, it is possible to interpret significant instrumental differences between cultivars or between cooking processes, providing equivalent analytical methods of analysis. In accordance with previous work, some significant differences in pulp hardness observed between cultivars during cooking (Ngalani & Tchango Tchango, 1998) would probably be detected by the panel.

#### Predicting sensory texture and sweetness with physicochemical parameters

Firmness, chewiness, mealiness, moistness and sweetness of boiled plantain were well predicted by the three chemical parameters, that is dry matter and soluble solid contents, and titratable acidity (Table 6). The models explained more than 85% of these attributes, and the quality of the predictions was sufficient to score sensory attributes with an error of less than 0.7 on a 0–10 point scale. To our knowledge, this is the first time that such predictions have been established on cooking banana.

**Table 6:** Prediction of sensory texture with boiled physicochemical parameters

Sensory attribute	Regression equation	Performance of calibration		Validation robustness	Sensitivity analysis
		*R* ^2^	RMSEC	RMSEV	
Firmness	Y = −1.09–0.40*SSC + 0.27*DMC	0.88	0.69	1.02	4.4 °Brix
Chewiness	Y = −1.27–0.26*SSC + 0.21*DMC	0.85	0.52	0.73	5.1 °Brix
Stickiness	Y = −3.08 + 0.25*TA	0.35	0.73	1.24	
Mealiness	Y = 7.60–0.33*SSC − 0.42*TA	0.93	0.61	0.87	4.7 °Brix
Moist	Y = 0.71 + 0.52*SSC	0.93	0.53	1.14	2.5 °Brix
Sweetness	Y = 0.52 + 0.50*SSC	0.87	0.73	0.90	3.7 °Brix

Calibration samples = 27; Validation samples = 8; DMC: dry matter content; SSC: soluble solids content; TA: titratable acidity. The sensitivity analysis provided the minimum instrumental difference required to ensure a perceptible sensory difference (*P* < 0.1). The table lists the difference only for the first prediction parameter and only for the best predictions.

As expected, sweetness was mainly predicted by soluble solid contents, confirming earlier results (Harker *et al*., 2002b; Colaric *et al*., 2005). An increase in the value of soluble solid contents of a Brix of 4° led to a 2‐point increase in sweetness, which was sufficient for the panellists to perceive a significant difference (*P* < 0.1).

Soluble solid contents played a major role in predicting firmness, chewiness, mealiness and moistness with an indirect impact. Indeed, soluble solid contents are an indicator of the degree of ripening, and, as such, inform on the rate of hydrolysed starch and solubilised cell walls (Prabha & Bhagyalakshmi, 1998). A higher rate of solubilised cell walls is expected especially after cooking, thereby reducing mechanical properties (especially firmness and chewiness) (Qi *et al*., 2000). As soluble solids are a consequence of ripening, gelatinisation of starch and cell wall degradation are induced by cooking. To predict firmness and chewiness, soluble solid contents were associated with dry matter content, which is known to influence the textural attributes of cooked starchy products (Ngalani & Tchango Tchango, 1998; Thygesen *et al*., 2001). Concerning mealiness (and conversely moistness), in raw fruits, it was previously suggested that peach pulp mealiness is a consequence of the degradation of cell wall pectin resulting in a gel‐like texture (Infante *et al*., 2009). The role of starch is likely to be critical for the texture of the pulp (Gibert *et al*., 2010) and during the ripening process (Shiga *et al*., 2011). Thus, it seems important to draw upon additional research on the characterisation of starch during cooking and ripening for the comprehension of the underlying mechanisms. Soluble solid contents of more than 4° Brix were needed to detect a difference in firmness, chewiness and mealiness, but only a Brix of 2.5° for moistness. We are unable to explain the poor prediction of adhesiveness (*R* ^2^ = 0.35, RMSEC = 0.73) by titratable acidity, as was previously the case in dessert bananas (Bugaud *et al*., 2013).

## Conclusion

The present study enabled us to describe the sensory diversity of boiled plantains from Ivory Coast. Robust predictors of boiled plantain firmness, chewiness, mealiness, moistness and sweetness were identified using easy‐to‐measure physicochemical and textural tests. The success of the predictions is probably partially due to our methodology, plus carefully monitoring temperature while the banana pulps were cooling. The proposed method could serve as a reference for further investigations into cooked products tasted at an optimal sensorial temperature. The identification of the instrumental predictors of the sensory quality of boiled plantain will enable breeders to screen new cooking hybrids earlier in selection schemes.

## Author contribution

**Hermann Antonin Kouassi:** Conceptualization (equal); Data curation (lead); Formal analysis (equal); Investigation (equal); Methodology (equal); Resources (supporting); Visualization (equal); Writing‐original draft (equal); Writing‐review & editing (equal). **Emma Fernande Assemand:** Funding acquisition (lead); Supervision (equal). **Olivier Gibert:** Conceptualization (supporting); Formal analysis (equal); Methodology (equal); Supervision (equal); Validation (equal); Visualization (equal); Writing‐original draft (equal); Writing‐review & editing (equal). **Julien Ricci:** Data curation (equal); Formal analysis (equal); Software (equal); Writing‐original draft (supporting). **ISABELLE MARAVAL:** Formal analysis (equal); Methodology (equal); Supervision (equal); Writing‐review & editing (supporting). **Deless Edmond Fulgence Thiemele:** Resources (lead). **Christophe Bugaud:** Conceptualization (lead); Data curation (equal); Formal analysis (equal); Investigation (equal); Methodology (lead); Project administration (lead); Supervision (lead); Validation (lead); Visualization (lead); Writing‐original draft (equal); Writing‐review & editing (lead).

## Conflict of interest

There is no conflict of interest.

## Ethical approval

Ethics approval was not required for this research.

### Peer review

The peer review history for this article is available at https://publons.com/publon/10.1111/ijfs.14765.

## Data Availability

Research data are not shared.
